# 2D–EM clustering approach for high-dimensional data through folding feature vectors

**DOI:** 10.1186/s12859-017-1970-8

**Published:** 2017-12-28

**Authors:** Alok Sharma, Piotr J. Kamola, Tatsuhiko Tsunoda

**Affiliations:** 1 Center for Integrative Medical Sciences, RIKEN Yokohama, Yokohama, 230-0045 Japan; 2 CREST, JST, Yokohama, 230-0045 Japan; 3 Institute for Integrated and Intelligent Systems, Griffith University, 170 Kessels Rd, Nathan, QLD 4111 Australia; 4 Medical Research Institute, Tokyo Medical and Dental University, Tokyo, 113-8510 Japan; 5 School of Engineering and Physics, University of the South Pacific, Laucala Bay Rd, Suva, Fiji

**Keywords:** EM algorithm, Feature matrix, Small sample size, Transcriptome, Methylome, Cancer, Phenotype clustering

## Abstract

**Background:**

Clustering methods are becoming widely utilized in biomedical research where the volume and complexity of data is rapidly increasing. Unsupervised clustering of patient information can reveal distinct phenotype groups with different underlying mechanism, risk prognosis and treatment response. However, biological datasets are usually characterized by a combination of low sample number and very high dimensionality, something that is not adequately addressed by current algorithms. While the performance of the methods is satisfactory for low dimensional data, increasing number of features results in either deterioration of accuracy or inability to cluster. To tackle these challenges, new methodologies designed specifically for such data are needed.

**Results:**

We present 2D–EM, a clustering algorithm approach designed for small sample size and high-dimensional datasets. To employ information corresponding to data distribution and facilitate visualization, the sample is folded into its two-dimension (2D) matrix form (or feature matrix). The maximum likelihood estimate is then estimated using a modified expectation-maximization (EM) algorithm. The 2D–EM methodology was benchmarked against several existing clustering methods using 6 medically-relevant transcriptome datasets. The percentage improvement of Rand score and adjusted Rand index compared to the best performing alternative method is up to 21.9% and 155.6%, respectively. To present the general utility of the 2D–EM method we also employed 2 methylome datasets, again showing superior performance relative to established methods.

**Conclusions:**

The 2D–EM algorithm was able to reproduce the groups in transcriptome and methylome data with high accuracy. This build confidence in the methods ability to uncover novel disease subtypes in new datasets. The design of 2D–EM algorithm enables it to handle a diverse set of challenging biomedical dataset and cluster with higher accuracy than established methods. MATLAB implementation of the tool can be freely accessed online (http://www.riken.jp/en/research/labs/ims/med_sci_math or http://www.alok-ai-lab.com/).

**Electronic supplementary material:**

The online version of this article (10.1186/s12859-017-1970-8) contains supplementary material, which is available to authorized users.

## Background

The cost of molecular profiling and recruiting large cohort of patients is often a prohibitive factor which results in many biomedical datasets having much higher number of features (or dimensions) *d* larger than sample number *n* (i.e., *d* >> *n*). This leads to a problem usually referred to as the small sample size (SSS) problem, and make it challenging to employ many state-of-the-art clustering algorithms to group the samples appropriately. Many clustering methods are based on maximum-likelihood approach or employ covariance information [1, 2]. However, when SSS problem exists, the covariance of samples becomes singular (or ill posed) and it is difficult to effectively utilize it in the application of clustering algorithms. This restricts us to the approaches which mainly employ norm distance (e.g. Euclidean norm) or centroid of samples to categorize samples into various clusters. Examples for such kind of algorithms are k-means or hierarchical clustering (which employs norm distance to build a dendrogram) [2].

In the literature, k-means clustering algorithm received widespread attention and has been used in a range of biological applications. The underlying functionality of many of the recent tools used in multiomics data analysis (iCluster, and iClusterPlus [3]) or cancer analysis (ConsensusCluster (CC) and CCPlus [4, 5]) was built using k-means. Though this type of method has been widely applied in the literature due to its easiness and appropriate level of clustering accuracy, it does not cluster based on data distribution as covariance information has not been employed. If we can gather more information from a limited amount of data then the clustering performance can be improved. This would have consequences in findings of biological sciences, especially in disease diagnosis or cancer subtypes analysis, multiomics data studies and population stratification [6].

A number of clustering algorithms other have been emerged in the literature. Here we briefly summarize exemplary methods. 1) Algorithms are developed using criteria functions, such as a) sum-of-squared error; b) scattering; c) related minimum variance; d) trace; e) determinant; and, f) invariant criterion [1, 7]; 2) clustering following iterative optimization [8–10]; 3) hierarchical clustering algorithms [11–14]; some conventional hierarchical-based algorithms are, single-linkage [15], complete-linkage [16], median-linkage [17], weighted average linkage [18] and ward linkage [19]. Single linkage (SLink) agglomerative hierarchical approach [15] combines clusters which are nearest to each other and applies Euclidean distance to quantity the nearness between the two neighboring groups. This method is sensitive to the positioning of samples, which sometimes causes an issue of a long chain (called the chaining effect). The hierarchical approach with complete linkage (CLink) [16] tries to reduce the chain effect by constructing groups using farthest-neighbor. However, it is susceptible to outliers. This problem can be overcome by applying average or median distance which was achieved in median linkage (MLink) hierarchical approach [17]. In the hierarchical weighted-average distance linkage (WLink) approach, group sizes are ignored while computing average distances. Consequently, smaller groups get larger weights during clustering [18]. In Ward’s linkage (Wa-Link), the clusters are joined based on an optimal value of an objective function. Similarly, in model-based hierarchical clustering [20, 21] an objective function is used. The method presented in [20] is based on the Bayesian analysis and uses multinomial likelihood function and Dirichlet priors. The approach in [21] optimizes the distance between two Gaussian mixture models. 4) Clustering is carried by Bayes classifier [22–26]; 5) by maximum likelihood in an iterative fashion [27–30]. In general, maximum likelihood can be computed via analytical procedure, grid search, hill-climbing procedure or EM algorithm [27, 31–35]; 6) spectral clustering use spectrum of similarity matrix to perform dimensionality reduction before conducting clustering [36], 7) non-negative matrix factorization (NNMF) [37] has also been used for clustering [38–40] and has been useful in handling high-dimensional data; and, 8) support vector clustering (SVC) became popular in recent literature [41–47]. However, its computational complexity is quite high and occasionally it fails to discover meaningful groups [14]. In general, for many applications clustering techniques constructed on maximum likelihood and Bayes approach are still the favored over support vector clustering. Maximum likelihood methods require differential calculus techniques or gradient search to estimate parameters. However, Bayes methods usually require solving complex multi-dimensional integration to reach to the solution. Since Bayes estimation methods has very high computational requirements [1], we prefer maximum likelihood in this paper.

Though many clustering methods have been developed in the literature for various applications [48–54], the problem of achieving a reasonable level of accuracy for high dimensional data still persists. Many of these algorithms fail to perform when the number of features is gradually increased and becomes huge in comparison with the number of samples [55–62]. Many methods that rely on data distribution, suffers from high dimensionality as such case create the problem of singularity of covariance matrix. Therefore, methods based on norm distance (e.g. Euclidean) or centroid based distance prevail in these situations. This is the usual case for many biological applications where generating additional samples is cost prohibitive. In order to deal with the dimensionality issue, in general either feature transformation or feature selection is applied to reduce (or transform) the data into a parsimonious space before executing clustering operation. This has its own advantages and disadvantages. Inspired by this drawback, we focus on developing a method that can easily and efficiently perform clustering on high dimensional data.

We propose a novel way of handing the data that precedes clustering. A sample (in a vector form) is reformed into a matrix form through a filtering process that simultaneously facilitates more straightforward visualization. This is a critical stage of this concept, as this reformation process can retain a significant amount of useful information for clustering that could otherwise be difficult to capture. Furthermore, we extended EM algorithm to estimate maximum likelihood for samples which appears in the matrix form (i.e. feature matrix) in contrast to the conventional methods which take input samples as feature vectors.

The novel method, which we named 2D–EM, has two steps. The first, filtering part produces a feature matrix for a sample while the subsequent clustering part is based on a modified EM algorithm that is capable of accepting these feature matrices as input. The maximum likelihood estimate via EM algorithm has been modified such that it can consider input as feature matrix instead of feature vector. The details of the method are given in the later section. We observed a significant improvement over many clustering algorithms over a number of transcriptome and methylome datasets evaluated in this study. We first present an overview of the maximum likelihood estimate via EM algorithm and then present our proposed 2D–EM clustering algorithm.

## Methods

### Overview of maximum likelihood estimate via EM algorithm

Here we briefly present the summary of the maximum likelihood via EM algorithm for clustering [1, 27, 63]. Suppose a *d*-dimensional sample set is described as *χ* = {x_1_, x_2_, …, x_*n*_} with *n* unlabelled samples. Let number of clusters be defined as *c*. Let the state of the nature or class label for *j*th cluster *χ*_*j*_ (for *j* = 1, …, *c*) be depicted as *ω*_*j*_. Let θ = {*μ*, Σ} be any unknown parameter (representing mean *μ* and covariance Σ). Then the mixture density would be

1 $$ p\left({\mathrm{x}}_k|\uptheta \right)=\sum \limits_{j=1}^cp\left({\mathrm{x}}_k|{\omega}_j,{\uptheta}_j\right)P\left({\omega}_j\right) $$

where *p*(x_*k*_| *ω*_*j*_, θ_*j*_) is the conditional density, θ = {θ_*j*_} (for *j* = 1…*c*), x_*k*_ ∈ *χ* and *P*(*ω*_*j*_) is the a priori probability. The log likelihood can be given by joint density

2 $$ L=\log p\left(\chi |\uptheta \right)=\log \prod \limits_{k=1}^np\left({\mathrm{x}}_k|\uptheta \right)=\sum \limits_{k=1}^n\log p\left({\mathrm{x}}_k|\uptheta \right) $$

If the joint density *p*(*χ*| θ) is differentiable w.r.t. to θ then from Eqs. 1 and 2

3 $$ {\nabla}_{\uptheta_i}L=\sum \limits_{k=1}^n\frac{1}{p\left({\mathrm{x}}_k|\uptheta \right)}{\nabla}_{\uptheta_i}\left[\sum \limits_{j=1}^cp\left({\mathrm{x}}_k|{\omega}_j,{\uptheta}_j\right)P\left({\omega}_j\right)\right] $$

where ${\nabla}_{\uptheta_i}L$ is defined as the gradient of *L* w.r.t. to θ_*i*_. If θ_*i*_ and θ_*j*_ are independent parameters and assume a posteriori probability is

4 $$ P\left({\omega}_i|{\mathrm{x}}_k,\uptheta \right)=\frac{p\left({\mathrm{x}}_k|{\omega}_i|{\uptheta}_i\right)P\left({\omega}_i\right)}{p\left({\mathrm{x}}_k|\uptheta \right)} $$

then from Eq. 4, we can observe that $\frac{1}{p\left({\mathrm{x}}_k|\uptheta \right)}=\frac{P\left({\omega}_i|{\mathrm{x}}_k,\uptheta \right)}{p\left({\mathrm{x}}_k|{\omega}_i,{\uptheta}_i\right)P\left({\omega}_i\right)}$. Substituting this value in Eq. 3 and since for any function *f*(*x*) its derivative *∂* log *f*(*x*)/*∂x* can be given as 1/*f*(*x*). *f* ^'^(*x*). We have

5 $$ {\nabla}_{\uptheta_i}L=\sum \limits_{k=1}^nP\left({\omega}_i|{\mathrm{x}}_k,\uptheta \right){\nabla}_{\uptheta_i}\log p\left({\mathrm{x}}_k|{\omega}_i|{\uptheta}_i\right) $$

If distribution of the data is normal Gaussian and *θ*_*i*_ = {*μ*_*i*_, Σ_*i*_} then we can employ Eq. 5 to find E-step and M-step of EM algorithm to find maximum likelihood estimate ${\widehat{\uptheta}}_i$. The solution be achieved by.

*E-step*

$$ {\phi}_{ik}=P\left({\omega}_i|{\mathrm{x}}_k,\mu, \Sigma \right) $$

*M-step*

6 $$ {\pi}_i=\frac{1}{n}\sum \limits_{k=1}^nP\left({\omega}_i|{\mathrm{x}}_k,\mu, \Sigma \right) $$

7 $$ {\mu}_i=\frac{\sum_{k=1}^n{\phi}_{ik}{\mathrm{x}}_k}{\sum_{k=1}^n{\phi}_{ik}} $$

8 $$ {\Sigma}_i=\frac{\sum_{k=1}^n{\phi}_{ik}\left({\mathrm{x}}_k-{\mu}_i\right){\left({\mathrm{x}}_{\mathrm{k}}-{\mu}_i\right)}^T}{\sum_{k=1}^n{\phi}_{ik}} $$

where *π*_*i*_ is the a priori probability, *μ*_*i*_ ∈ ℝ^*d*^ and Σ_*i*_ ∈ ℝ^*d* × *d*^. For a normal distribution case, *ϕ*_*ik*_ can be expressed as

9 $$ {\phi}_{ik}=\frac{p\left({\mathrm{x}}_k|{\omega}_i,{\mu}_i,{\Sigma}_i\right){\pi}_i}{\sum_{j=1}^cp\left({\mathrm{x}}_k|{\omega}_j,{\mu}_j,{\Sigma}_j\right){\pi}_j}=\frac{{\left|{\Sigma}_i\right|}^{-1/2}\exp \left[-\frac{1}{2}{\left({\mathrm{x}}_k-{\mu}_i\right)}^T{\Sigma}_i^{-1}\left({\mathrm{x}}_k-{\mu}_i\right)\right]{\pi}_i}{\sum_{j=1}^c{\left|{\Sigma}_j\right|}^{-1/2}\exp \left[-\frac{1}{2}{\left({\mathrm{x}}_k-{\mu}_j\right)}^T{\Sigma}_j^{-1}\left({\mathrm{x}}_k-{\mu}_j\right)\right]{\pi}_j} $$

For every iteration check whether $L=\sum \limits_{k=1}^n\log \sum \limits_{j=1}^c{\pi}_jp\left({\mathrm{x}}_k|{\omega}_j,{\mu}_j,{\Sigma}_j\right)$ is converging. At the convergence of *L* this procedure yields maximum likelihood estimate ${\hat{\theta}}_i=\left\{{\hat{\mu}}_i,{\hat{\Sigma}}_i\right\}$ (for *i* = 1, 2, …, *c*).

As it can be observed from the above procedure, the maximum likelihood estimate is possible if the inverse of covariance matrix exists. For high dimensional data (where samples are relatively lower), the computation of maximum likelihood estimate becomes difficult as covariance matrix becomes singular.

### 2D–EM clustering methodology

**Fig. 1:**
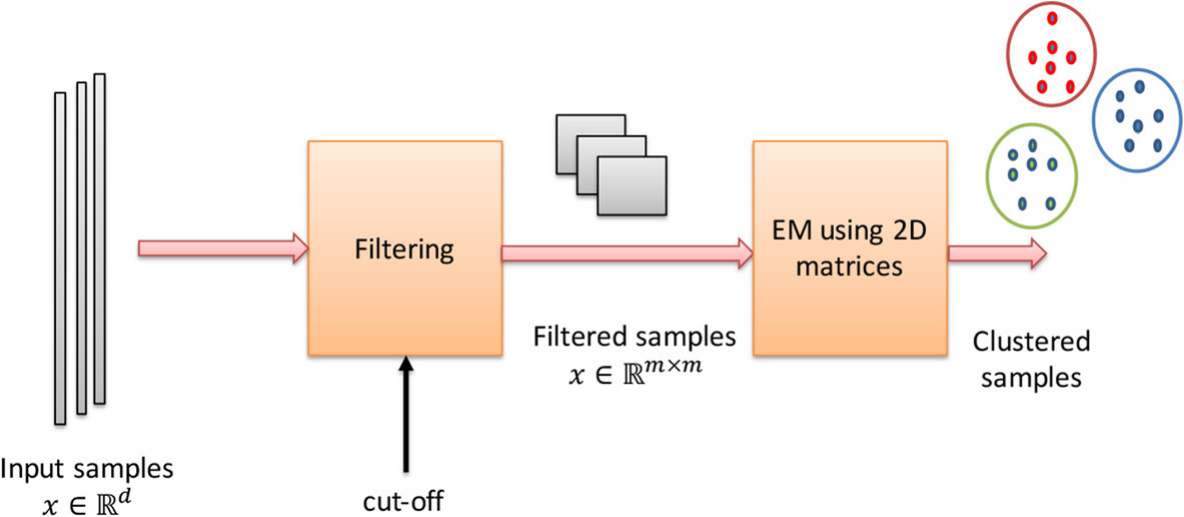
An illustration of 2D–EM clustering algorithm

In this section, we describe our proposed 2D–EM clustering algorithm. In order to overcome the dimensionality problem, we propose to fold a feature vector x ∈ ℝ^*d*^ into a matrix form *X* ∈ ℝ^*m* × *q*^ (where *mq* ≤ *d*, number of rows of a feature matrix *X* is denoted as *m* whereas number of columns is denoted as *q*). Thereafter, we find maximum likelihood estimate using EM algorithm for matrices. The 2D–EM algorithm has two main components: 1) filtering step and 2) clustering step. In the filtering part, a feature vector x is reformed into its matrix form or feature matrix *X*. In the clustering step, feature matrices (or samples in the form of *X*) are clustered. Figure 1 illustrates the overall procedure of 2D–EM clustering algorithm.

Input samples are first processed through a filter where each sample is formed as a matrix. Thereafter, these feature matrices are sent to the clustering process.

Here we first describe the clustering part of 2D–EM algorithm for feature matrices to obtain maximum likelihood estimate. Let a sample *X*_*k*_ ∈ ℝ^*m* × *q*^ (where *m* ≤ *q*) be formed from x_*k*_ ∈ ℝ^*d*^ by a filtering process (to be discussed later). We define the mean *M* ∈ ℝ^*m* × *q*^ and covariance *C* ∈ ℝ^*m* × *m*^ for feature matrices.

The class-conditional density for a feature matrix *X*_*k*_ can be described as,

10 $$ p\left({X}_k|{\omega}_i,{\theta}_i\right)=\frac{1}{{\left(2\pi \right)}^{m\times \boldsymbol{q}}{\left|{C}_i\right|}^{1/2}}\exp \left(-\frac{1}{2} trace\left({\left({X}_k-{M}_i\right)}^T{C}_i^{-1}\left({X}_k-{M}_i\right)\right)\right) $$

The derivative of likelihood function can be obtained in a similar way as that of maximum likelihood estimate and it comes similar to Eq. 5 as

11 $$ {\nabla}_{\uptheta_i}L=\sum \limits_{k=1}^nP\left({\omega}_i|{\mathrm{X}}_k,\uptheta \right){\nabla}_{\uptheta_i}\log p\left({\mathrm{X}}_k|{\omega}_i,{\uptheta}_i\right) $$

This fortunately simplifies the derivations of maximum likelihood estimate for feature matrices and the 2D–EM procedure can be described as.

*2D E-step*

$$ {\phi}_{ik}=P\left({\omega}_i|{\mathrm{X}}_k,M,C\right) $$

*2D M-step*

12 $$ {\pi}_i=\frac{1}{n}\sum \limits_{k=1}^nP\left({\omega}_i|{\mathrm{X}}_k,M,C\right) $$

13 $$ {M}_i=\frac{\sum_{k=1}^n{\phi}_{ik}{X}_k}{\sum_{k=1}^n{\phi}_{ik}} $$

14 $$ {C}_i=\frac{\sum_{k=1}^n{\phi}_{ik}\left({\mathrm{X}}_k-{M}_i\right){\left({\mathrm{X}}_{\mathrm{k}}-{M}_i\right)}^T}{\sum_{k=1}^n{\phi}_{ik}} $$

In a similar way, for a normal distribution case, *ϕ*_*ik*_ can be expressed as

15 $$ {\phi}_{ik}=\frac{p\left({X}_k|{\omega}_i,{M}_i,{C}_i\right){\pi}_i}{\sum_{j=1}^cp\left({X}_k|{\omega}_j,{M}_j,{C}_j\right){\pi}_j}=\frac{{\left|{C}_i\right|}^{-1/2}\exp \left[-\frac{1}{2} trace\left[{\left({\mathrm{X}}_k-{M}_i\right)}^T{C}_i^{-1}\left({\mathrm{X}}_k-{M}_i\right)\right]\right]{\pi}_i}{\sum_{j=1}^c{\left|{C}_j\right|}^{-1/2}\exp \left[-\frac{1}{2} trace\left[{\left({\mathrm{X}}_k-{M}_j\right)}^T{C}_j^{-1}\left({\mathrm{X}}_k-{M}_j\right)\right]\right]{\pi}_j} $$

Again, for every iteration it can be observed if likelihood *L* is converging.

It can be seen from Eq. 14 that covariance matrix is no longer of *d* × *d* size, however, it is reduced to size *m* × *m*. Since *m* ^2^ ≤ *d*, theoretically we can say that the size of covariance matrix is reduced to the square root (or less) of the data dimensionality. This reduction is achieved without performing linear or non-linear transformation (of data). Furthermore, this enables us to use Eq. 15 effectively as singularity problem of *C*_*i*_ matrix is reduced at least by the square root of the data dimensionality.

Next, we discuss the filtering process. The objective of this process is to form a sample x ∈ ℝ^*d*^ into a matrix *X* ∈ ℝ^*m* × *q*^ form. For convenience, here we use *q* = *m*; i.e., size of *X* would be *m* × *m*. This filtering process has two parts: 1) feature selection, and 2) matrix arrangement.

In the feature selection part, we perform ANOVA to find *p*-values for each of the features and then retain the top *m* ^2^ features. Here we have used *p*-values as a prototype to filter genes or features. However, one can use any other scheme, e.g. regression methods (logistic regression, linear regression, Poisson regression, Lasso etc.) depending upon the application or specific type of data used. Since we do not know the class labels of data, we need to find temporary class labels to compute *p*-values for features. Therefore, to obtain *p*-values, we perform hierarchical clustering to find *c* clusters. Thereafter, from the known labels we can compute *p*-values which will help us to remove some features. This process will give us a feature vector $y\in {\mathrm{\mathbb{R}}}^{m^2}$ where *m* ^2^ ≤ *d* and features in *y* is arranged corresponding to the low to high *p*-values.

In the matrix arrangement part, we arrange *y* to get a feature matrix *X* ∈ ℝ^*m* × *m*^. To arrange features in *X* systematically so that any two samples can be compared without having a conflict, we applied a simple rule. We computed the mean *μ*_*y*_ from all *y* samples and then arranged features of *μ*_*y*_ in ascending order. Thereafter, we arranged features of *y* corresponding to the order of features of *μ*_*y*_. This allows us to put features in a common format for all the samples. Next, we reshape $y\in {\mathrm{\mathbb{R}}}^{m^2}$ so that it becomes *X* ∈ ℝ^*m* × *m*^.

The value of *m* can be computed as follows. First, the cut-off for *p*-values will reduce dimensions from *d* to *h* (where *h* ≤ *d*). Then *m* can obtained as $m=\left[\sqrt{h}\right]$, where $\left[\sqrt{h}\right]\le \sqrt{h}$ and [˙] is an integer; i.e., *m* is an integer smaller or equal to $\sqrt{h}$. The arrangement of feature matrix process is summarized in Table 1. The filtering process is summarized in Table 1.

**Table 1:** Arrangement of features into *m* × *m* matrix

Feature Selection
1. Given x ∈ *χ* in a *d*-dimensional space.
2. Perform hierarchical clustering on all samples x to find temporary class labels.
3. Using these class labels find *p*-values for all the *d* features.
4. Find *m* by placing a threshold or cut-off on *p*-values (e.g. cut-off for *p*-values could be 0.01).
5. Retaining the top *m* ^2^ features will give us a sample $y\in {\mathrm{\mathbb{R}}}^{m^2}$, where all *y* samples form a sample set $Y\in {\mathrm{\mathbb{R}}}^{m^2\times n}$.
Matrix arrangement
6. Compute mean $$ {\mu}_y=\frac{1}{n}\sum \limits_{y\in Y}y $$.
7. Arrange features of *μ*_*y*_ in ascending order and note the indices.
8. Arrange features of *y* by following the indices from step 7.
9. Reshape a sample *y* to a matrix *X* ∈ ℝ^*m* × *m*^.

**Fig. 2:**
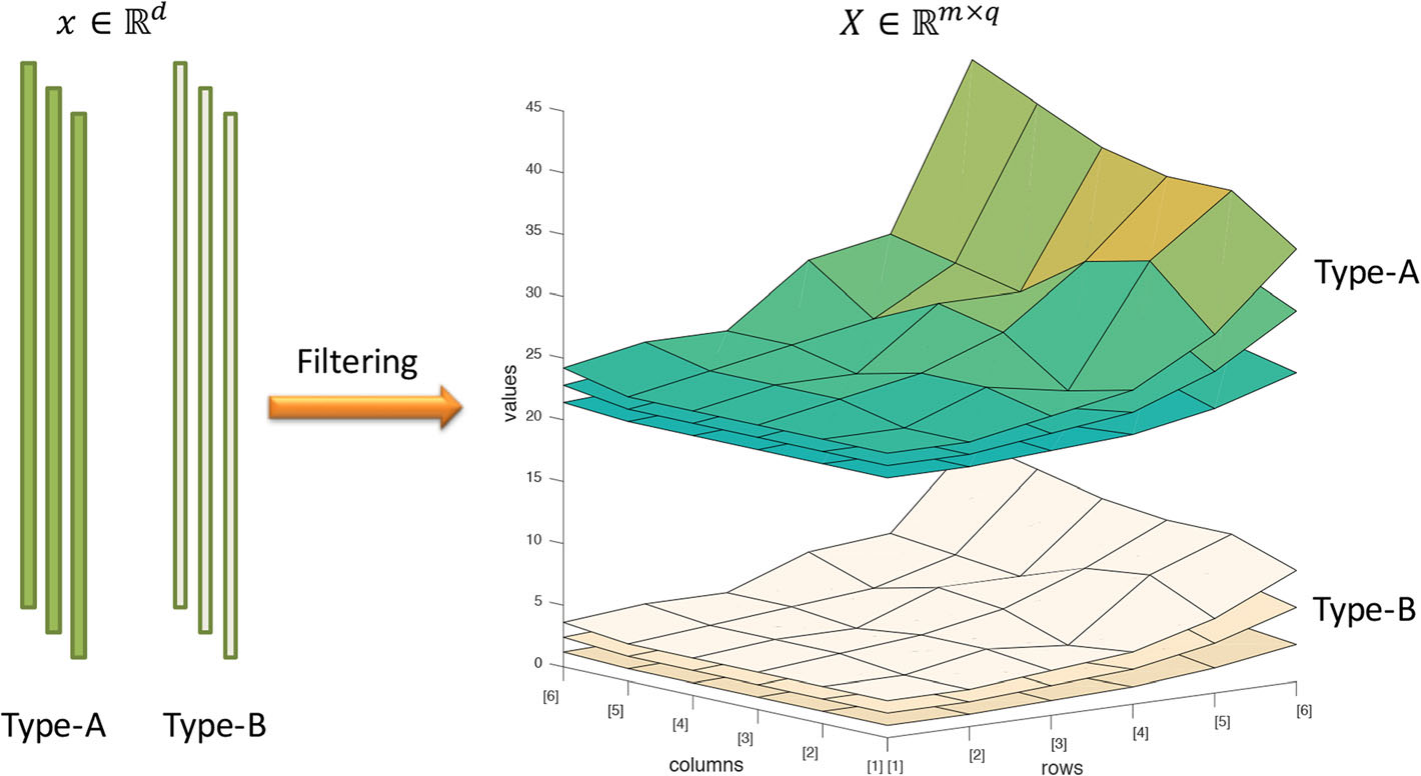
Visualization of high dimensional data

It is also possible to visualize feature matrix *X* and can be compared with other samples to see the difference or similarity. Figure 2 provides an illustration of visualization of high dimensional data. A feature vector *x* ∈ ℝ^*d*^ is constructed as a feature matrix *X* ∈ ℝ^*m* × *q*^ through the filtering process (as described in Table 1). For this illustration, two different groups of samples (Type-A and Type-B) which were difficult to visualize in ℝ^*d*^ space, are shown on ℝ^*m* × *q*^ space. The visualization of feature matrix is more meaningful in the matrix space.

**Fig. 3:**
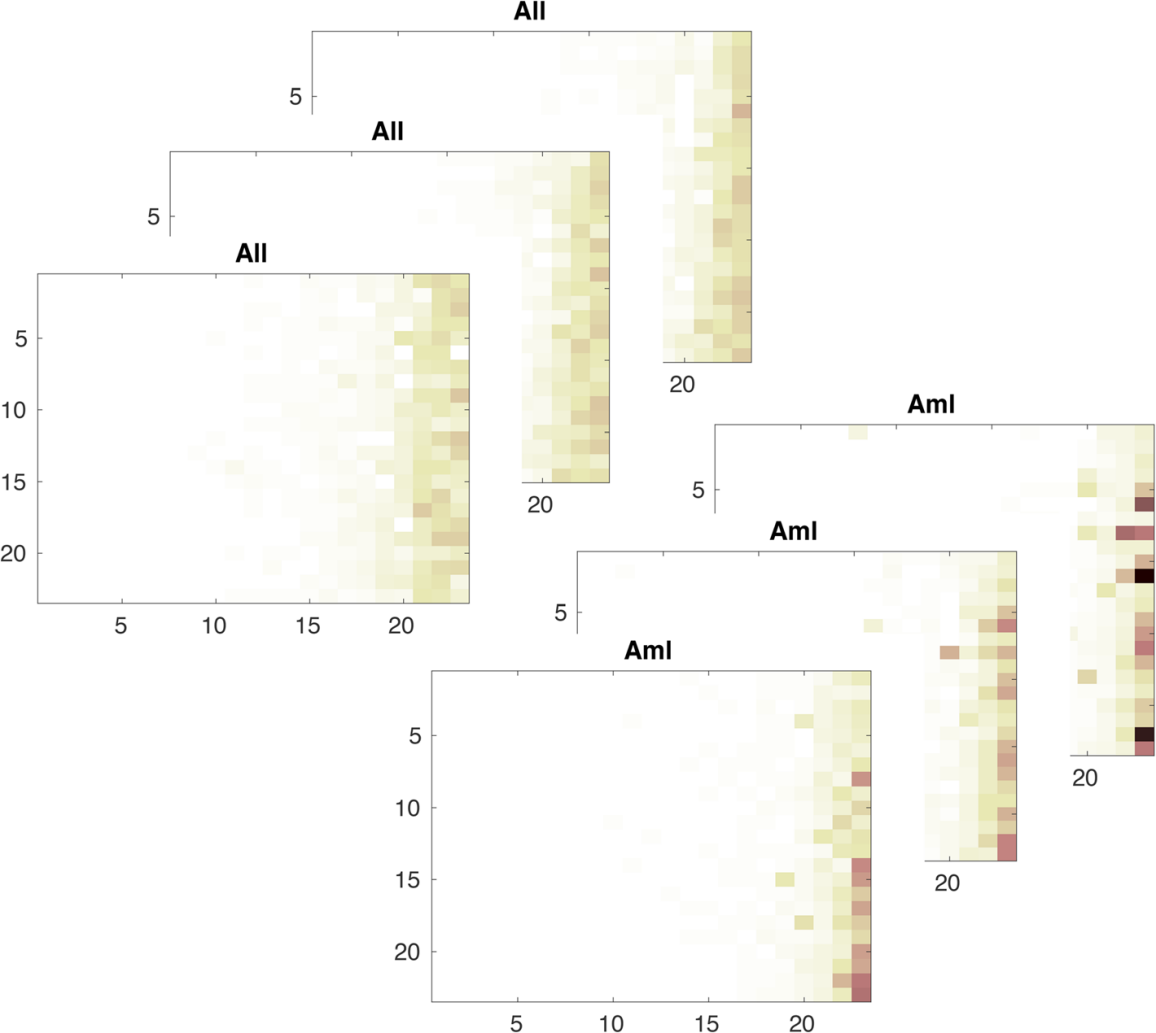
Visualization of feature matrix: *acute lymphoblastic leukemia* (ALL) vs. *myeloid leukemia* (AML). An ALL sample or feature vector *x* ∈ ℝ^*d*^ is transformed to feature matrix *X* ∈ ℝ^*m* × *m*^ using the procedure outlined in Table 1. These feature matrices are shown at top right side of the figure. Similarly, a sample of AML is also transformed to feature matrix and shown at bottom right side of the figure

To further demonstrate this with transcriptome data, we consider six samples from ALL dataset (data used in this paper are described later in Section 3.1). These samples are randomly picked for this illustration. Three samples belong to cluster *acute lymphoblastic leukemia* (ALL) and the other three samples belong to cluster *acute myeloid leukemia* (AML). The number of features (or dimensions) of these samples is 7129 and it is impossible to visualize data in 7129-dimensional space. However, using filtering (from Table 1) we can visualize each sample as a matrix (see Fig. 3). Just by looking at the patterns of these feature matrices, it can be observed that samples from ALL are different from that of AML. The patterns of AML feature matrices have high intensity (or shades) at specific locations compared to the patterns of ALL feature matrices. This reformation of sample from vector to matrix form assist in data visualization and pattern recognition. Similarly, it would also improve the power of detection for a clustering method provided if the method was designed well to utilize this information.

## Results and discussion

In order to verify the performance of 2D–EM clustering algorithm, we employed 6 transcriptome and 2 methylome datasets described below. We used several clustering algorithms and employed Rand score [64] and adjusted Rand index [65] as a performance measure to compare the clustering algorithms in this study. The Rand scoring reflects how well the group labels were reproduced using unlabeled data, and a high score build confidence in the methods ability to detect novel groups in novel data for which no phenotype labels are available. These are well known measures to gauge the performance of clustering algorithm [66]. The results are described in the ‘Clustering on transcriptome data’ and ‘Clustering on methylome data’ sections.

### Biomedical datasets

Acute leukemia dataset [67]: contains DNA microarray gene expressions of acute leukemia samples. Two kinds of leukemia are provided, namely acute myeloid leukemia (AML) and acute lymphoblastic leukemia (ALL). It consists of 25 AML and 47 ALL bone marrow samples over 7129 probes. The features are all numeric having 7129 dimensions.

Small round blue-cell tumor (SRBCT) dataset [68]: has 83 samples of the RNA expression profiles of 2308 genes. The tumors are the Ewing family of tumors (EWS), Burkitt lymphoma (BL), neuroblastoma (NB), and rhabdomyosarcoma (RMS). The dataset consists of 29, 11, 25 and 18 samples of EWS, BL, RMS and NB, respectively.

MLL Leukemia [69]: has three groups ALL, AML leukemia and mixed lineage leukemia (MLL). The dataset contains 20 MLL, 24 ALL and 28 AML. The dimensionality is 12,582.

ALL subtype dataset [70]: contains 12,558 gene expressions of acute lymphoblastic leukemia subtypes. It has 7 groups namely E2A-PBX1, BCR-ABL, MLL, hyperdiploid >50 chromosomes ALL, TEL-AML1, T-ALL and other (contains diagnostic samples that did not fit into any of the former six classes). Samples per group are 27, 15, 20, 64, 79, 43 and 79, respectively.

Global cancer map (GCM) [71]: has 190 samples over 14 classes with 16,063 gene expressions.

Lung Cancer [72]: contains gene expression levels of adenocarcinoma (ADCA) and malignant mesothelioma (MPM) of the lung. In total, 181 tissue samples with 12,533 genes are given where 150 belongs to ADCA and 31 belongs to MPM.

Gastric Cancer [73]: 32 pairs of gastric cancer and normal (adjacent) tissue were profiled using Illumina Infinium HumanMethylation27 BeadChip. 27,579 CpG sites were interrogated at a single-nucleotide resolution. Both Beta- and M-values statistics were calculated from the methylated and unmethylated signals as described in [74].

Hepatocellular Carcinoma [75]: 20 pairs of hepatocellular tumor and their non-tumor tissue counterparts were evaluated using the same platform (27,579 CpG sites) and processed in the same manner as in Gastric cancer dataset.

A summary of the transcriptome and methylome datasets is depicted in Table 2. It is evident from the table that the number of features (genes or CpG site methylation state) is much larger than the number of samples for all the datasets. This creates SSS problem in all the cases.

**Table 2:** Transcriptome and methylome datasets

Datasets	Features	Samples	Classes
ALL Leukemia	7129	72	2
SRBCT	2308	83	4
MLL	12,582	72	3
ALL Subtype	12,558	327	7
GCM	16,063	198	14
Lung Cancer	12,553	181	2
Gastric Cancer	27,579	64	2
Hepatocellular Carcinoma	27,579	40	2

### Clustering on transcriptome data

In this subsection, we show the performance of various clustering methods in terms of Rand score [64] over 6 transcriptome datasets. Rand score shown here represents an average taken from over 10 repetitions. Rand score is similar to clustering accuracy and it value lies between 0 and 1. We also used adjusted Rand index [65], which assumes the generalized hypergeometric model. Adjusted Rand index can attain wider range of values than Rand score.

### Rand and adjusted Rand scores

For 2D–EM clustering algorithm we use 0.01 as a cut-off during the filtering process (the reasoning behind selecting this particular cut-off is described in section ‘Effect of using filter’). Table 3 depicts the Rand score analysis and Table 4 shows adjusted Rand index. We have employed several clustering methods for comparison. These methods are k-means, hierarchical clustering methods (SLink, CLink, ALink, MLink, Ward-Link and Weighted-Link), spectral clustering, mclust [76] and NNMF clustering. For k-means and hierarchical clustering methods, packages from MATLAB software were used. For NNMF clustering method, package provided by ref. [38] was used. For spectral clustering, package provided by ref. [77] was used. In all the cases, only data was provided with the number of cluster information.

**Table 3:** Rand score (highest values are highlighted as bold faces)

Method	SRBCT	ALL	MLL	ALL subtype	GCM	Lung cancer
K-means	0.58	0.53	0.78	0.64	0.84	0.72
CLink	0.30	0.49	0.54	0.52	0.71	0.70
ALInk	0.30	0.56	0.35	0.51	0.38	0.71
Ward-Link	0.44	0.56	0.78	0.53	0.84	0.80
Weighted-Link	0.30	0.52	0.51	0.52	0.61	0.71
Mlink	0.30	0.55	0.35	0.48	0.54	0.71
Spectral Clustering	0.39	0.51	0.56	0.63	0.55	0.71
NNMF Clustering	**0.66**	0.50	0.74	0.64	0.83	0.63
Mclust	0.51	0.50	0.61	0.30	0.83	0.57
2D–EM	0.65	**0.62**	**0.80**	**0.78**	**0.87**	**0.84**

**Table 4:** Adjusted Rand index (highest values are highlighted as bold faces)

Method	SRBCT	ALL	MLL	ALL subtype	GCM	Lung cancer
Kmeans	0.13	0.03	0.47	0.15	0.19	0.22
CLink	0.00	−0.03	0.13	0.00	0.09	−0.02
ALInk	0.00	0.05	0.00	−0.01	0.01	−0.01
Wa-Link	0.00	0.09	0.51	0.00	0.17	0.41
Wt-Link	0.00	−0.03	0.08	0.00	0.07	−0.01
Mlink	0.00	0.02	0.00	−0.01	0.08	−0.01
Spectral Clustering	−0.02	0.02	0.02	0.00	0.07	−0.01
NNMF Clustering	0.18	0.00	0.42	0.11	0.17	0.26
Mclust	−0.02	−0.01	0.21	−0.01	0.09	0.05
2D–EM	**0.19**	**0.23**	**0.57**	**0.26**	**0.22**	**0.62**

It can be observed from Table 3 that for SRBCT dataset, NNMF clustering is showing 0.66 Rand score followed by 0.65 of 2D–EM. However, adjusted Rand index (Table 4) for SRBCT is better for 2D–EM. For all other datasets 2D–EM is performing the best in terms of Rand score and adjusted Rand index (Table 3 and Table 4).

For an instance, we can observe that from Table 3, 2D-EM scored highest Rand score of 0.62 followed by ALink (0.56) and Ward-link (0.56) on ALL dataset. For MLL k-means and Ward-link scored 0.78 and 2D–EM was able to score 0.80. In the case of ALL subtype, 2D–EM scored 0.78 followed by k-means (0.64) and NNMF (0.64). For GCM, 2D–EM got 0.87 followed by k-means (0.84) and Ward-link (0.84). For Lung Cancer, Ward-link scored 0.80 and 2D–EM reached 0.84. We can also observe that spectral clustering underperforming when the dimensionality is large. Similarly, many clustering methods (not reported here) did not provide results due to high number of features.

Similarly, we can see from Table 4 that 2D–EM is way ahead on ALL dataset by attaining 0.23 adjusted Rand index followed by second best of 0.09 by Ward-link. For MLL dataset, 2D–EM scored 0.57 followed by Ward-link (0.51) and mclust (0.51). In case of ALL subtype and GCM datasets, 2D–EM (0.26, 0.22) is followed by k-means (0.15, 0.19). For Lung dataset, 2D–EM scored 0.62 followed by mclust (0.36).

The improvement (in terms of Rand score and adjusted Rand index) of 2D–EM over the best performing existing method has been depicted in Table 5. It can be noticed that the best percentage improvement for Rand score compared to the best performing clustering method is 21.9%. Similarly, the best percent improvement in terms of adjusted Rand index is 155.6%.

**Table 5:** Percentage improvement of 2D–EM clustering method over other existing clustering methods

Parameter	SRBCT	ALL	MLL	ALL subtype	GCM	Lung cancer
Rand Score	−1.5	10.7	2.6	21.9	3.6	5.0
Adjusted Rand Index	5.6	155.6	11.8	73.3	21.1	51.2

### Average performance

**Fig. 4:**
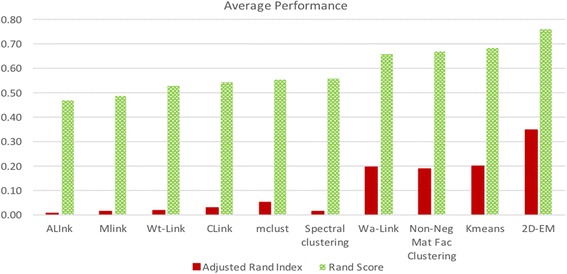
Comparison of average performance (in terms of Rand score and Adjusted Rand index)

We have also compared the average of Rand score and adjusted Rand index over all the datasets used. The comparison is depicted in Fig. 4. The comparison of average performance is interesting. It can be seen that k-means clustering algorithm performs quite reasonably for high dimensional data. Several clustering algorithms have been proposed after k-means algorithm, yet for high dimensional data the average performance has not been improved. Apart from k-means algorithm, Ward-Link hierarchical clustering, NNMF clustering, mclust and spectral clustering were able to attain reasonable level of performance. The 2D–EM clustering algorithm was able to attain 11.4% improvement on Rand score, and 75.0% improvement on adjusted Rand index over the best performing method. Therefore, it can be concluded that in all the cases 2D–EM was able to achieve very promising results.

### Effect of using filter

The 2D–EM clustering algorithm uses a filtering step to arrange a feature vector into a feature matrix. We want to analyze the effect of applying this filter to other clustering algorithms. In order to perform this analysis, we preprocess data to retain top *m* ^2^ features by filtering before executing other clustering algorithms (note samples are not reshaped in matrix form for other methods as this would require changing the mathematics of algorithms). The detailed results are given in Additional file 1: it can be observed from Tables S1, S2, S3 and S4 that after applying filter for other clustering methods, the performance doesn’t improve significantly. Therefore, the evidence of bias due to filtering process is weak.

### Effect of variable cut-off

**Fig. 5:**
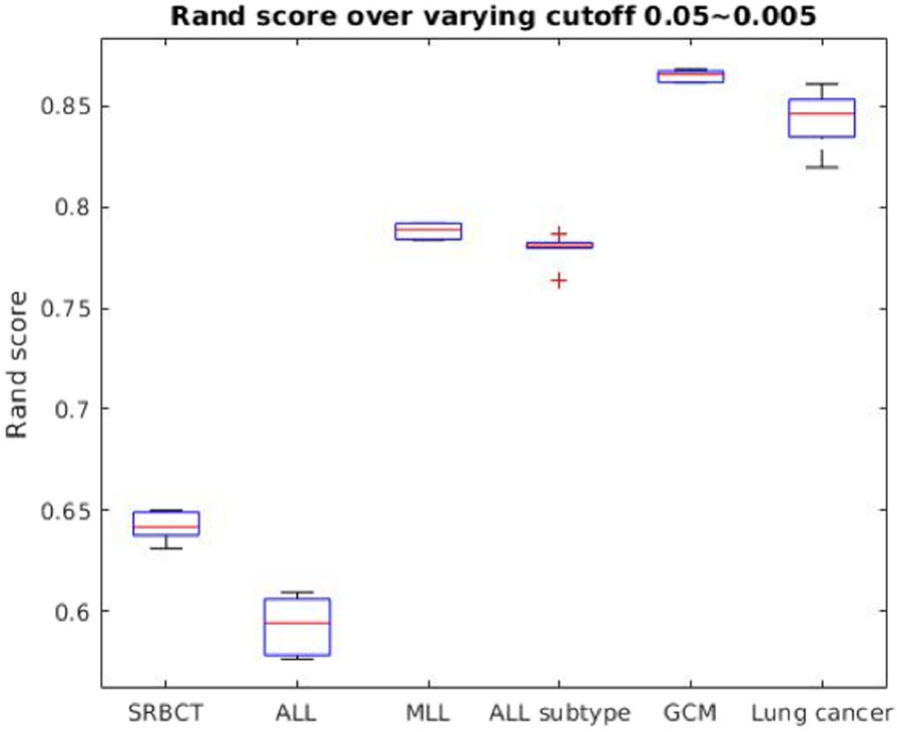
Box plot showing the effect of changing cut-off value for 2D–EM clustering algorithm

In order to illustrate the effect of changing the cut-off value for the 2D–EM clustering algorithm, we varied cut-off value from 0.05 to 0.005 and noted the Rand score over 10 repetitions. The box-plot with the corresponding results is shown in Fig. 5. It can be noticed from Fig. 5, that varying cut-off value over a range (0.05~0.005) does not significantly change the Rand score of the algorithm. Therefore, the selection of 0.01 cut-off value in the previous experiment is not a sensitive choice.

### Clock time

The processing (clock) time of 2D–EM clustering algorithm when run on Linux platform (Ubuntu 14.04 LTS, 64 bits) having 6 processors (Intel Xeon R CPU E5–1660 v2 @ 3.70GHz) and 128 GB memory per repetition is as follows. On SRBCT dataset, 2D–EM clustering algorithm took 11.4 s. Similarly, on ALL, MLL, ALL subtype, GCM and Lung datasets, processing time were 8.7 s, 47.1 s, 286.5 s, 358.2 s and 82.0 s, respectively. Therefore, for all the transcriptome datasets used in this study, the processing time for 2D–EM clustering algorithm was within 6 mins.

### Consistency

**Fig. 6:**
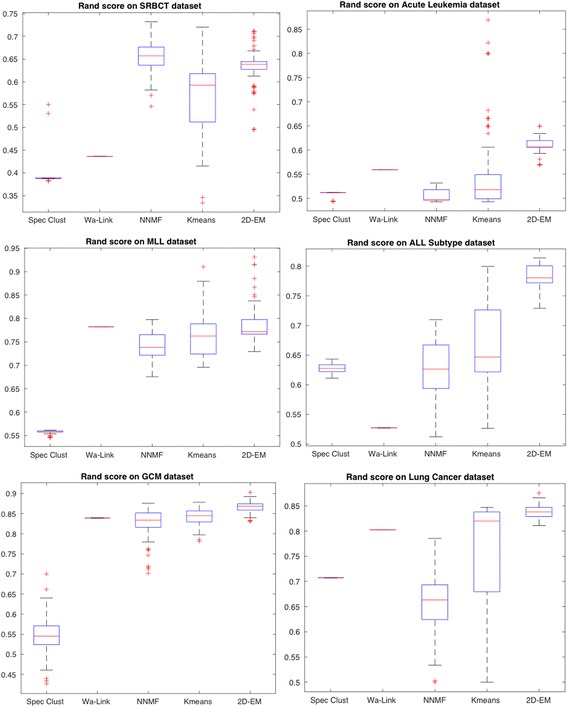
Rand score of five best performing methods over 100 runs

**Fig. 7:**
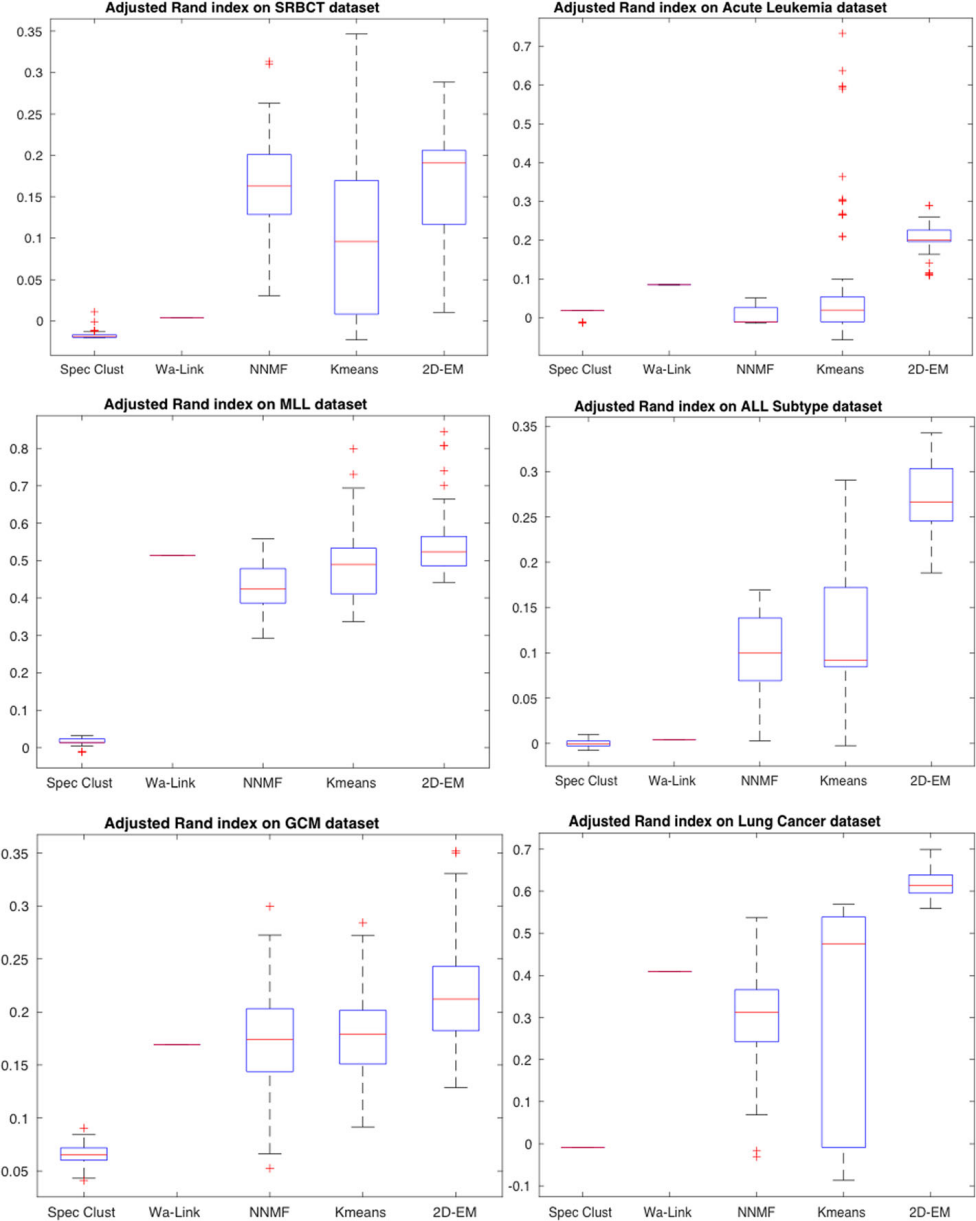
Adjusted Rand index over 100 runs

To verify the consistency or stability of 2D–EM clustering algorithm, we employed top five performing clustering algorithms and obtain boxplots of Rand score and adjusted Rand index over all the transcriptome datasets used. The results are derived from over 100 runs. Figure 6 depicts boxplot of Rand score of 5 best methods (spectral clustering, Wa-Link, NNMF, k-means and 2D–EM). It can be observed that on SRBCT dataset NNMF is showing superior performance followed by 2D–EM clustering algorithm. However, on all the remaining 5 datasets (ALL, MLL, ALL Subtype, GCM and Lung Cancer), 2D–EM is outperforming all the clustering methods. Similarly, adjusted Rand index was computed on the same datasets and shown in Fig. 7. Again, 2D–EM clustering methodology outperformed all the clustering methods in terms of adjusted Rand index.

### Clustering on methylome data

To show the utility of 2D–EM methodology we evaluated two additional datasets of clinical relevance. While in previous examples we showed commonly used transcriptome data, the full understanding of biological phenomena can only be achieved by considering multiple genomics ‘layers’. To this end, we compared the Rand score and adjusted Rand index on DNA methylation data. Epigenetic modifications measured in those datasets are known to affect a wide range of biological processes and diseases phenotypes [78]. As we are approaching the era of personalized medicine, clustering of different genomic components will continue to rise in prominence.

**Fig. 8:**
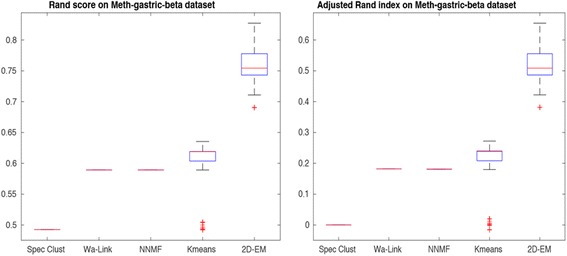
Rand score and adjusted Rand index on Gastric cancer methylation data (Beta-values)

**Fig. 9:**
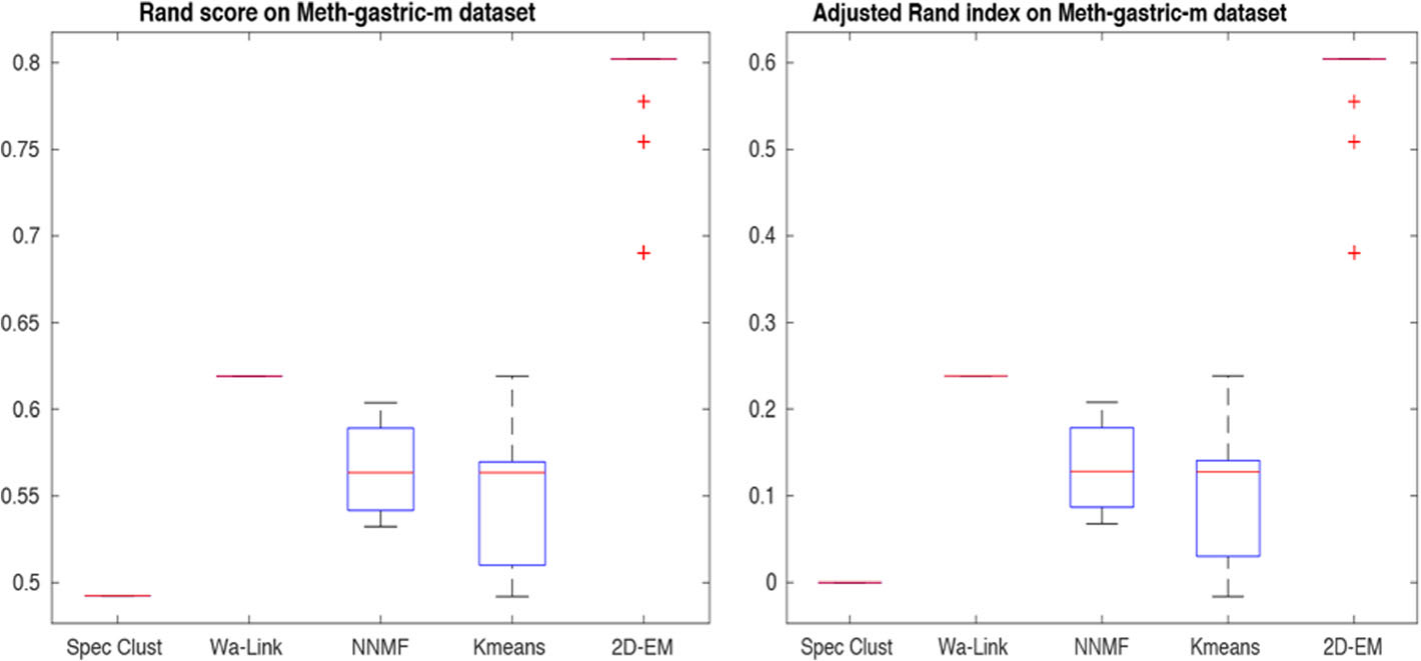
Rand score and adjusted Rand index on Gastric cancer methylation data (M-values)

For this purpose, we compared the performance of the best 5 methods (selected based on performance with transcriptome data). These methods are spectral clustering, Ward-link hierarchical clustering, NNMF, k-means and 2D–EM. Figure 8 depicts Rand score and adjusted Rand index on Gastric cancer methylation data using Beta-values over 100 runs. It can be clearly observed that 2D–EM is outperforming other methods even when different type of data is tested. Similarly, Fig. 9 shows the results on Gastric data using alternative M-values, again for over 100 runs. Again 2D–EM accurately recreated the phenotype labels.

**Fig. 10:**
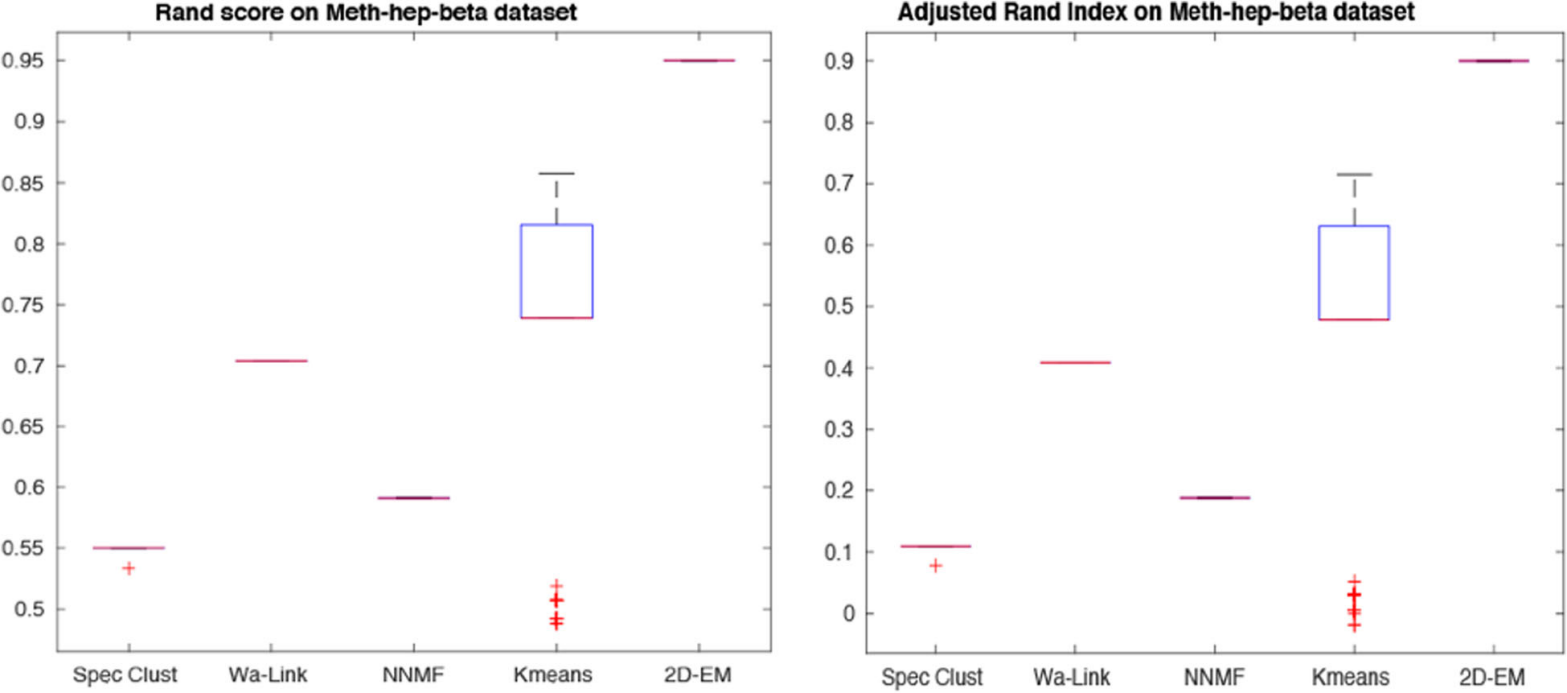
Rand score and adjusted Rand index on Hepatocellular carcinoma (Beta-values)

**Fig. 11:**
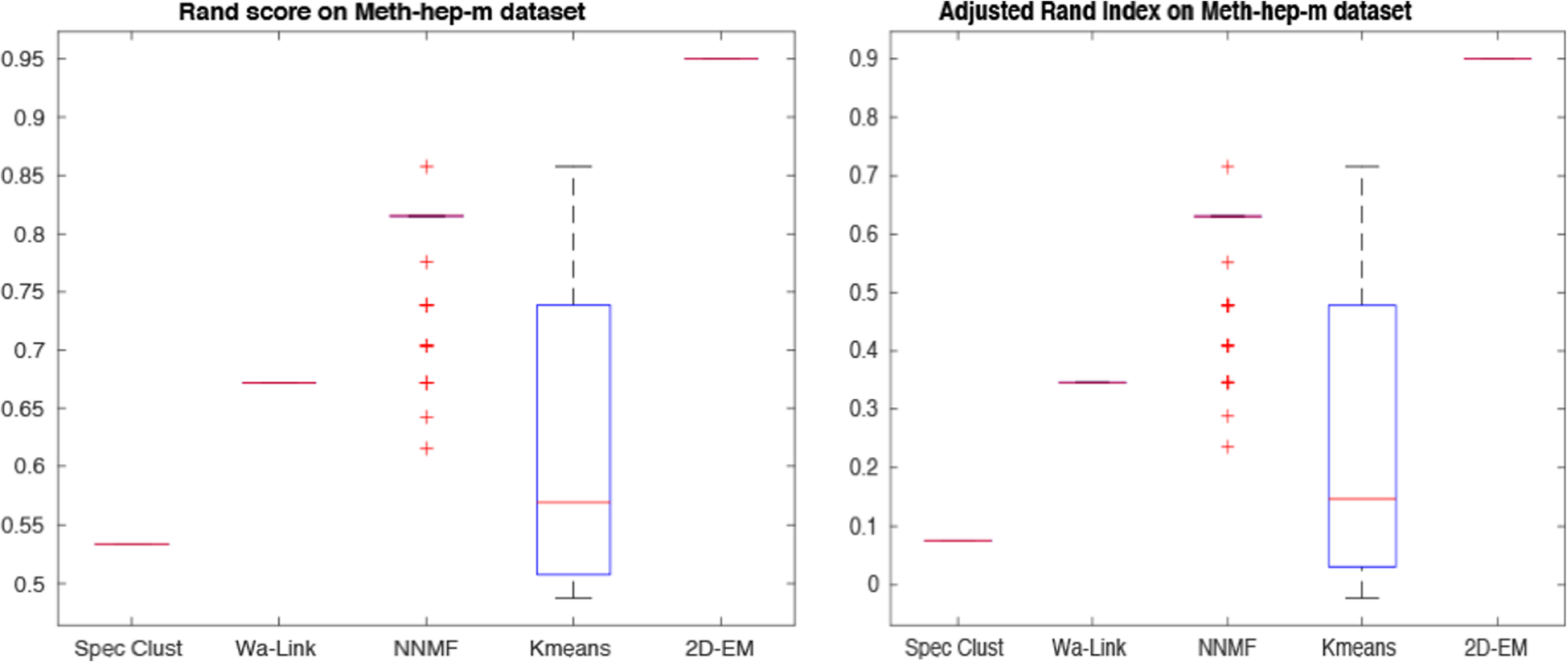
Rand score and adjusted Rand index on Hepatocellular carcinoma (M-values)

We have also carried out tests on Hepatocellular carcinoma data, with results shown in Figs. 10 and 11 for Beta- M-values respectively. Similar to the Gastric dataset, 2D–EM is achieving very promising results for both Beta- and M-values.

## Conclusions

By looking at the nature of data readily found biological sciences, in this work we proposed 2D–EM clustering algorithm. This methodology clusters a given data in two steps. In the first step, it reformats a feature vector to a matrix form and, in the second part, it conducts the clustering. The advantage of 2D–EM algorithm is that it can perform clustering at high dimensional space (compared to the number of samples) by effectively incorporating data distribution information via its covariance matrix. The proposed method avoids the singularity issue by folding a feature vector into a feature matrix. This reduces the dimensionality from *d* to less than $\sqrt{d}$. Thereby, distribution information along with distance information can be used to cluster a sample. The algorithm was compared to several existing clustering algorithms over a number of transcriptome and methylome datasets, and managed to accurately reproduce the phenotype labels that were hidden from the analysis. MATLAB package of 2D–EM clustering algorithm can be found by visiting our website (http://www.riken.jp/en/research/labs/ims/med_sci_math or http://www.alok-ai-lab.com). In the future, we will investigate ways to extend the present method to Bayesian estimation and hierarchical methods.

## Additional file

Additional file 1: In this file the bias of using filtering process is analyzed. Here, we analyzed the effect of applying the filter (which was used for 2D–EM algorithm) to other clustering algorithms. We preprocess data to retain top *m* ^2^ features. The *m* ^2^ values for all datasets at 0.01 cut-off were as follows: 1156 (SRBCT), 529 (ALL), 6084 (MLL), 1444 (ALL subtype), 15,129 (GCM) and 5625 (Lung Cancer). Then clustering algorithms are applied to see the difference in performance (both in Rand score and adjusted Rand index). **Table S1** and **Table S2** show the Rand score and adjusted Rand score when filtering step is applied. **Table S3** and **Table S4** show the variations in Rand score and adjusted Rand score after filtering compared to before filtering process. (DOCX 25 kb)

## Abbreviations

2D: Two-dimension
ADCA: Adenocarcinoma
ALL: Acute lymphoblastic leukemia
AML: Acute myeloid leukemia
BL: Burkitt lymphoma
CLink: Complete Linkage
EM: Expectation-maximization
EWS: Ewing family of tumors
GCM: Global Cancer Map
MLink: Median Linkage
MLL: Mixed lineage leukemia
MPM: Malignant mesothelioma
NB: Neuroblastoma
NNMF: Non-negative matrix factorization
RMS: rhabdomyosarcoma
SLink: Single linkage
SSS: Small sample size
SVC: Support vector clustering
Wa-Link: Ward’s linkage
WLink: Weighted average distance linkage
