# Supplementary material for: 2D–EM clustering approach for high-dimensional data through folding feature vectors
Source: BMC Bioinformatics. 2017 Dec 28;18(Suppl 16):547. doi: 10.1186/s12859-017-1970-8 (PMC5751765; doi:10.1186/s12859-017-1970-8)
Supplement: Additional file 1: — In this file the bias of using filtering process is analyzed. Here, we analyzed the effect of applying the filter (which was used for 2D–EM algorithm) to other clustering algorithms. We preprocess data to retain top m 2 features. The m 2 values for all datasets at 0.01 cut-off were as follows: 1156 (SRBCT), 529 (ALL), 6084 (MLL), 1444 (ALL subtype), 15,129 (GCM) and 5625 (Lung Cancer). Then clustering algorithms are applied to see the difference in performance (both in Rand score and adjusted Rand index). Table S1 and Table S2 show the Rand score and adjusted Rand score when filtering step is applied. Table S3 and Table S4 show the variations in Rand score and adjusted Rand score after filtering compared to before filtering process. (DOCX 25 kb) [file 12859_2017_1970_MOESM1_ESM.docx]

**Additional File 1**

The bias of using filtering process can be analyzed. We preprocess data to retain top $m^{2}$ features. The $m^{2}$ values for all datasets at $0.01$ cut-off were as follows: 1156 (SRBCT), 529 (ALL), 6084 (MLL), 1444 (ALL subtype), 15129 (GCM) and 5625 (Lung Cancer). Then clustering algorithms are applied to see the difference in performance (both in Rand score and adjusted Rand index). Table S1 shows Rand score of all the clustering methods when filtering step is applied. Similarly, Table S2 depicts adjusted Rand index.

Table S1 - Rand score by using filter for all the clustering algorithms (highest values are highlighted as bold faces).

| **Methods** | **SRBCT** | **ALL** | **MLL** | **ALL subtype** | **GCM** | **Lung Cancer** |
| --- | --- | --- | --- | --- | --- | --- |
| Filter + k-means | 0.55 | 0.53 | 0.76 | 0.54 | 0.84 | 0.82 |
| Filter + CLink | 0.30 | 0.52 | 0.56 | 0.52 | 0.73 | 0.80 |
| Filter + ALInk | 0.30 | **0.54** | 0.35 | 0.50 | 0.39 | 0.80 |
| Filter + Ward-Link | 0.45 | 0.52 | **0.77** | 0.53 | **0.87** | 0.80 |
| Filter + Weighted-Link | 0.30 | 0.50 | 0.53 | 0.52 | 0.62 | 0.71 |
| Filter + Mlink | 0.30 | 0.52 | 0.74 | 0.49 | 0.53 | 0.71 |
| Filter + Spec. clustering | 0.50 | 0.49 | 0.53 | 0.63 | 0.47 | 0.68 |
| Filter + NNMF clustering | **0.58** | 0.53 | 0.75 | 0.62 | 0.84 | **0.92** |
| Filter + mclust | 0.56 | **0.54** | 0.54 | **0.73** | 0.83 | 0.53 |

Table S2 - Adjusted Rand index by using filter for all the clustering algorithms (highest values are highlighted as bold faces).

| **Methods** | **SRBCT** | **ALL** | **MLL** | **ALL subtype** | **GCM** | **Lung Cancer** |
| --- | --- | --- | --- | --- | --- | --- |
| Filter + k-means | **0.07** | 0.04 | 0.48 | 0.01 | 0.18 | 0.47 |
| Filter + CLink | 0.00 | 0.04 | 0.15 | 0.00 | 0.10 | 0.41 |
| Filter + ALInk | 0.00 | 0.01 | 0.00 | -0.01 | 0.01 | 0.41 |
| Filter + Ward-Link | -0.01 | 0.02 | **0.49** | 0.00 | **0.23** | 0.41 |
| Filter + Weighted-Link | 0.00 | -0.02 | 0.11 | 0.00 | 0.06 | -0.01 |
| Filter + Mlink | 0.00 | -0.02 | 0.48 | -0.01 | 0.08 | -0.01 |
| Filter + Spec. clustering | 0.00 | -0.04 | -0.01 | 0.01 | 0.06 | -0.04 |
| Filter + NNMF clustering | **0.07** | 0.04 | 0.45 | 0.09 | 0.18 | **0.80** |
| Filter + mclust | **0.07** | **0.08** | 0.01 | **0.15** | 0.09 | 0.03 |

To observe how the scores changed after applying the filtering step, we can subtract scores obtained after filtering by scores of before filtering. These variations in Rand score and adjusted Rand index are summarised in Table S3 and Table S4.

Table S3 - Variation in Rand score after the application of filter (significant change highlighted as bold faces).

| **Methods** | **SRBCT** | **ALL** | **MLL** | **ALL subtype** | **GCM** | **Lung Cancer** |
| --- | --- | --- | --- | --- | --- | --- |
| Kmeans | -0.03 | 0.00 | -0.02 | -0.10 | 0.00 | 0.10 |
| CLink | 0.00 | 0.03 | 0.02 | 0.00 | 0.02 | 0.10 |
| ALInk | 0.00 | -0.02 | 0.00 | -0.01 | 0.01 | 0.09 |
| Wa-Link | 0.01 | -0.04 | -0.01 | 0.00 | 0.03 | 0.00 |
| Wt-Link | 0.00 | -0.02 | 0.02 | 0.00 | 0.01 | 0.00 |
| MLink | 0.00 | -0.03 | **0.39** | 0.01 | -0.01 | 0.00 |
| Spectral clustering | 0.11 | -0.02 | -0.03 | 0.00 | -0.08 | -0.03 |
| Non-Neg Mat Fac Clust | -0.08 | 0.03 | 0.01 | -0.02 | 0.00 | 0.29 |
| Mclust | 0.05 | 0.04 | -0.07 | **0.43** | 0.00 | -0.04 |

Table S4 - Variation in adjusted Rand index after the application of filter (significant change highlighted as bold faces).

| **Methods** | **SRBCT** | **ALL** | **MLL** | **ALL subtype** | **GCM** | **Lung Cancer** |
| --- | --- | --- | --- | --- | --- | --- |
| Kmeans | -0.06 | 0.01 | 0.01 | -0.14 | -0.01 | **0.25** |
| CLink | 0.00 | 0.07 | 0.02 | 0.00 | 0.01 | **0.43** |
| ALInk | 0.00 | -0.04 | 0.00 | 0.00 | 0.00 | **0.42** |
| Wa-Link | -0.01 | -0.07 | -0.02 | 0.00 | 0.06 | 0.00 |
| Wt-Link | 0.00 | 0.01 | 0.03 | 0.00 | -0.01 | 0.00 |
| MLink | 0.00 | -0.04 | **0.48** | 0.00 | 0.00 | 0.00 |
| Spectral clustering | 0.02 | -0.06 | -0.03 | 0.01 | -0.01 | -0.03 |
| Non-Neg Mat Fac Clust | -0.11 | 0.04 | 0.03 | -0.02 | 0.01 | **0.54** |
| Mclust | 0.09 | 0.09 | -0.20 | 0.16 | 0.00 | -0.02 |

It can be observed from Table S3 that even after applying filter for other clustering methods, the performance doesn’t improve significantly. For SRBCT, ALL, GCM and Lung Cancer datasets, the change in Rand score is minimal. For MLL, only MLink clustering algorithm has changed by 0.39 and for ALL subtype mclust has changed significantly. For SRBCT, ALL, ALL subtype and GCM datasets no significant change was observed. For Lung Cancer dataset, the change in adjusted Rand indexes for k-means, CLink, ALink and NNMF are observed. Overall, in 5 out of 54 cases, adjusted Rand score has varied significantly. This is around 9.2%, which can also be considered not significant.

Therefore, we can conclude that the strategy of applying filtering didn’t help other clustering algorithms. This is because even after filtering process the dimensionality of data is still very high which is deteriorating the performance of many clustering methods.

It is interesting to note that after performing filtering, the performance for many methods did not alter much. However, for 2D-EM the results were encouraging. This implies that the use of folding a feature vector into feature matrix form prior to clustering helps. This way we can avoid singularity issues of covariance matrix. If a feature vector is folded efficiently then it can provide distribution information along with distance information for clustering algorithms. This supports in improving the performance of clustering methods (which rely on covariance matrix).
